# Dedução, Indução e a Arte do Raciocínio Clínico na Educação Médica: Revisão Sistemática e Proposta Bayesiana

**DOI:** 10.36660/abc.20220405

**Published:** 2022-11-09

**Authors:** Marcos Roberto de-Sousa, Túlio Roberto Xavier de Aguiar

**Affiliations:** 1 Hospital das Clínicas Universidade Federal de Minas Gerais Belo Horizonte MG Brasil Hospital das Clínicas da Universidade Federal de Minas Gerais , Belo Horizonte , MG – Brasil; 2 Departamento de Filosofia Faculdade de Filosofia e Ciências Humanas Universidade Federal de Minas Gerais Belo Horizonte MG Brasil Departamento de Filosofia da Faculdade de Filosofia e Ciências Humanas – FAFICH – Universidade Federal de Minas Gerais (UFMG), Belo Horizonte , MG – Brasil

**Keywords:** Educação Médica, Resolução de Problemas, Aprendizagem, Tomada de Decisão Clínica, Revisão Sistemática, Teorema de Bayes, Medicina Baseada em Evidências

## Abstract

**Fundamento:**

O raciocínio clínico está no centro da prática médica e emaranhado em uma confusão conceitual.A teoria da dualidade da probabilidade permite analisar seus aspectos objetivos e subjetivos.

**Objetivos:**

Fazer revisão sistemática da literatura sobre o raciocínio clínico para tomada de decisão na educação médica e uma proposta chamada “Pensamento Conforme a Regra de Bayes” (PCRB).

**Métodos:**

A revisão sistemática foi realizada na base PubMed até a data de 27/02/2022, seguindo metodologia rigorosa, por pesquisador experiente em revisão sistemática. A proposta PCRB, apresentada na discussão, foi elaborada no trabalho de conclusão de graduação em Filosofia na Universidade Federal de Minas Gerais. Usou-se a insuficiência cardíaca como exemplo.

**Resultados:**

De 3340 artigos encontrados, incluíram-se 154 artigos: 24 tratando da condição de incerteza; 87 tratando de conceitos vagos (discussão de casos, heurística, lista de vieses cognitivos, escolha com sabedoria) incluídos sob o termo ‘arte’; e 43 discutindo a ideia geral de raciocínio indutivo ou dedutivo. PCRB oferece regras de coerência e reprodutibilidade, inferência sob incerteza e regra de aprendizado, e pode, por meio da perspectiva subjetiva sobre a probabilidade, incorporar aqueles termos vagos classificados como ‘arte’, bem como argumentos e evidências.

**Conclusões:**

A revisão sistemática mostra que o raciocínio é fundado na incerteza, predominantemente probabilístico; além de mostrar algumas possibilidades de erro do pensamento hipotético-dedutivo. O PCRB é um pensamento probabilístico em duas etapas que pode ser ensinado. A regra de Bayes é uma ferramenta linguística, uma regra geral de raciocínio, de diagnóstico, de comunicação científica e de revisão do conhecimento médico conforme novas evidências.

## Introdução

O raciocínio está no centro da prática médica, espalhado por diversas disciplinas e tradições, em meio a grande confusão conceitual. ^1^ O raciocínio se dá em um nível bioquímico, elétrico e magnético que não compreendemos muito bem, apesar dos avanços da neurociência, por ser pré-linguístico. ^2^ Por meio da expressão linguística, podemos ensinar raciocínios: lógico, matemático, e probabilístico, incluindo o “pensamento conforme a regra de Bayes” (PCRB).

“Medicina baseada em evidências” (MBE) descreve um movimento iniciado contra uma dependência excessiva de julgamento e experiência clínica na tomada de decisões de tratamento. ^3^ MBE é o uso consciente, explícito e criterioso das melhores evidências atuais na tomada de decisões sobre o cuidado de pacientes individuais. ^4^ Sob uma perspectiva dual da probabilidade, a MBE seria uma versão em que se valoriza as frequências nos ensaios clínicos e o raciocínio do médico seria a formação coerente de “graus de crença”, ^5^ regida pela teoria subjetiva da probabilidade e, basicamente, seguindo o PCRB. Este incorpora as evidências dentro de um contexto prévio, de forma que um ensaio clínico isolado não é suficiente para suplantá-lo.

O raciocínio lógico tende a ser dedutivo, determinístico e, por isto, é diferente do raciocínio probabilístico que é indutivo e fundado na incerteza (não determinístico). Faremos um ataque ao exagero do uso de raciocínios dedutivos e de evidências isoladas. Tanto os raciocínios dedutivos e argumentativos quanto as evidências podem ser incorporados ao PCRB. A motivação para este trabalho foi uma pesquisa filosófica ^5 - 8^ sobre o raciocínio realizada por um médico com experiência em ensino e pesquisa na área da saúde. O objetivo é realizar uma revisão sistemática da literatura sobre raciocínio e tomada de decisão na educação médica, bem como apresentar explicação e argumentos em defesa do PCRB, um tipo específico de raciocínio probabilístico.

## Métodos

A revisão sistemática foi feita seguindo diretrizes PRISMA. ^9^ No tesauro MeSH ( *Medical Subject Headings* ), ‘raciocínio clínico’ é um descritor subordinado ao termo ‘diagnóstico’. Já o descritor ‘tomada de decisão’ fica sob ‘processos mentais’. Neste descritor, ‘tomada de decisão’ é definida como “o processo de se fazer um julgamento intelectual seletivo quando apresentado a várias alternativas complexas de várias variáveis e geralmente de se definir um curso de ação ou uma ideia”. ‘Educação médica’ era a principal questão de interesse, então a seguinte busca reprodutível foi realizada:

(((“Education, Medical”[Majr]) AND ((“Clinical Reasoning”[Majr]) OR “Decision Making”[Majr]))) AND ((“1952/02/27”[Date - Entry] : “2022/02/27”[Date - Entry]))

As referências dos artigos foram utilizadas. Os critérios de inclusão foram artigos em inglês, alemão, português e espanhol; todos os tipos de publicação, com foco em educação para o raciocínio clínico, tomada de decisão, métodos de pensamento, estudos de caso. Os critérios de exclusão foram artigos que discutiam a tomada de decisão para escolhas na carreira médica, *marketing* médico, decisões do sistema de saúde, e questões não relacionadas ao raciocínio. Estudos com comparações utilizando questionários para especialistas, questionários para estudantes, resultados de estudos de caso estruturados, esquemas ou jogos sérios só interessavam quando discutiam argumentos a respeito de regras gerais de raciocínio.

A avaliação crítica da literatura médica incluiu o uso do PCRB, ^10^ porque esta já era uma proposta *a priori* . Após exclusões por título ou resumo usando o aplicativo RAYYAN, ^11^ a seleção dos artigos foi feita após primeira leitura do texto completo. Como o principal interesse estava nos argumentos dos autores em defesa de suas teorias sobre o raciocínio clínico, todos os artigos foram lidos uma segunda vez para análise dos argumentos; sendo divididos em três grupos definidos *a posteriori* : 1- incerteza; 2-conceitos vagos incluídos sob termo ‘arte’; e 3- a ideia geral de raciocínio. Este último grupo é o de maior interesse, mas considerou-se que os outros dois grupos oferecem argumentos relevantes.

A proposta PCRB foi elaborada por um pesquisador médico durante um trabalho de conclusão de curso de graduação em filosofia orientado por um experiente filósofo da ciência, avaliando o trabalho de Ian Hacking ^5 , 8^ e Donald Gillies ^7^ sobre as teorias filosóficas sobre a probabilidade e a regra de Bayes. Esta é uma probabilidade condicional da hipótese posterior dada a evidência, ou seja, uma revisão de probabilidades de cenários à luz de novas evidências ou informações, expressa por:


$$\mathrm{Pr}(H|E)=\frac{\mathrm{Pr}(H)\mathrm{Pr}(E|H)}{\mathrm{Pr}(H)\mathrm{Pr}(E|H)+\mathrm{Pr}(∼H)\mathrm{Pr}(E|∼H)}$$

Outra opção de fórmula: “ ***odds pós teste = razão de verossimilhança x odds pré teste*** ”. Sensibilidade, especificidade e razão de verossilhança são alternativas de medidas de acurácia. ^12^ Pr(E|H) é a sensibilidade e Pr(E|~H) = (1 – especificidade). Pr(H|E) é a revisão da hipótese (probabilidade pós-teste ou probabilidade do cenário posterior) com base na taxa base (Pr(H)) do cenário prévio (probabilidade pré-teste) e na acurácia da nova evidência ou informação. O cenário posterior tem uma probabilidade que é determinada pela probabilidade do cenário prévio (taxa base) e pela acurácia (também uma probabilidade) da nova evidência ou informação. Trata-se de uma combinação condicional de probabilidades em duas etapas temporalmente articuladas. Com base na teoria subjetiva da probabilidade, o estado de crença do médico é representado por uma função de probabilidade. A cada momento, seu estado de crença é atualizado com base nas informações recebidas da anamnese, exame clínico, exames complementares e também da literatura médica (que pode usar medidas de frequência menos subjetivas). Usa-se, na discussão, a insuficiência cardíaca para ilustrar as possibilidades de uso do PCRB no diagnóstico, prognóstico e escolha terapêutica.

## Resultados

**Figura 1:**
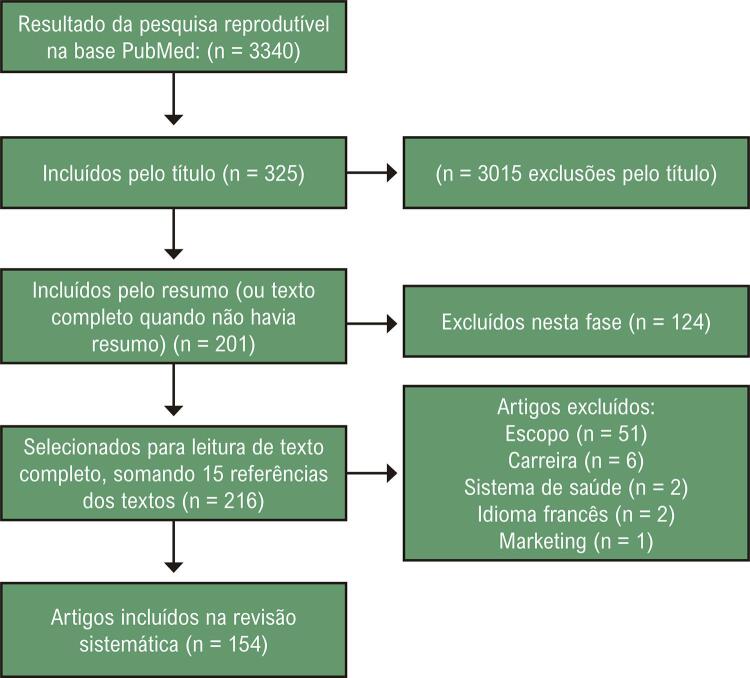
Fluxograma de seleção de estudos.

O fluxograma de seleção de artigos está na Figura 1 . De 3340 referências avaliadas, foram incluídos 154 artigos: 24 tratando da incerteza, 87 tratando de conceitos incluídos sob o termo ‘arte’ e 43 discutindo a ideia geral de raciocínio. Esses três grupos de artigos serão apresentados em três seções a seguir. Como seria inviável citar 154 referências neste artigo, por questões de espaço, optou-se por oferecer um Apêndice (https://bit.ly/3EMx5sp) com uma sucinta explicação da classificação e divisão dos artigos em grupos, com todas as referências.

### Seção 1: Incerteza

Havia 24 artigos baseados na ideia de que estudantes de medicina e médicos deveriam aprender a lidar com a ‘incerteza’ ( Tabela 1 ). O significado da palavra ‘incerteza’ inclui desde incerteza diagnóstica até incerteza sobre o próprio conhecimento do médico e a literatura científica. Esses artigos foram reunidos porque formam a base epistêmica do PCRB. A incerteza é a condição humana para o raciocínio contingente. Em vez de rejeitá-la, os médicos devem entender e aprender a lidar com a incerteza, por meio do uso de argumentos e evidências, em uma hierarquia que será apresentada na proposta final.

**Tabela 1:** Principais argumentos ou ideias nos 24 artigos que discutem a incerteza

Autor, Ano	Tipo de publicação	Argumento principal
Whitehorn, 1963	Opinião de especialista	Contra o determinismo, em favor da probabilidade e dos valores.
Elstein, 1982	Editorial	Uma proposta para lidar com a incerteza: "Mais tempo é gasto na computação e interpretação de qui-quadrados, testes T e outras técnicas inferenciais que nas estatísticas de revisão de opinião e tomada de decisão – a chamada abordagem Bayesiana – embora este último seja mais relevante para o trabalho diário da prática clínica (...). Os clínicos estão mais preocupados com a solidez das decisões tomadas em circunstâncias particulares do que com a solidez das inferências gerais. Para isso, a perspectiva Bayesiana será mais útil, e a instrução na lógica das decisões clínicas deve incorporá-la. "
Gunderman, 2005	Opinião de especialista	Uma proposta dialógica para lidar com a incerteza.
Nevalainen et al., 2009	22 diários de alunos	Escrita reflexiva como uma proposta de expressar e lidar com a incerteza.
Blanch et al., 2009	147 interações registradas de alunos com pacientes	Pesquisadores encontraram uma percepção negativa de estudantes de medicina que expressaram incerteza para seus pacientes. Foram analisados tipos de frases.
Charlin et al., 2010	Painéis com especialistas, moradores e estudantes	Uma proposta de uso de métodos de padronização para comparar os escores de examinadores individuais com os de um painel agregado, a fim de lidar com a incerteza.
Schwartz, 2011	Opinião de especialista	Uma proposta de ensinar a tomada de decisões como uma questão principal na medicina.
Hamui-Sutton et al., 2015	128 Residentes: entrevista e opinião de especialista	Uma avaliação abrangente de vários tipos de incerteza envolvida na prática médica.
Niedermier, 2016	Carta ao editor	Aponta para efeitos negativos da incerteza e observa a necessidade de treinamento para lidar com isso.
Simpkin et al., 2016	Opinião de especialista	Considerar “hipóteses” em vez de “diagnósticos”, abraçando a incerteza como atitude.
Cooke et al., 2017	594 estagiários	Estresse da incerteza e relutância em comunicar incerteza.
Cooke et al., 2017	Opinião de especialista	Uma proposta de abraçar a incerteza e aceitar mais de uma conduta possível para o problema apresentado.
Oferta, 2017	Opinião de especialista	Literatura, música, arte e humanidades como proposta aprendem a lidar com a incerteza.
Kim et al., 2018	Revisão	Proposta de estratégias para gestão da incerteza.
Simpkin et al., 2018	86 Entrevistas de residentes e opiniões de especialistas	Altos níveis de estresse devido à incerteza e baixos níveis de resiliência parecem estar associados à depressão e *burnout.*
Tonelli et al., 2019	Opinião de especialista	Abordagem filosófica da incerteza, incluindo metafísica, falibilismo e racionalidade epistêmica.
Davidson, 2019	Editorial	Um conjunto de recomendações para lidar com a incerteza na ciência médica: "Os autores devem ser adequadamente temperados em suas conclusões, usando linguagem que reconhece a incerteza quando apropriado. As conclusões devem ser influenciadas não apenas pelo valor p, mas também pelo tamanho do efeito e pelos limites dos intervalos de confiança de 95%."
Ying et al., 2019	70 residentes em uma pesquisa	O estudo sugeriu que aqueles que estão mais confortáveis com a incerteza podem experimentar maior satisfação no trabalho.
Stephens et al., 2020	Estudo qualitativo com 608 alunos	Incentivar educadores médicos a incorporar aspectos de tolerância da incerteza em ambientes curriculares e de aprendizagem.
Beck et al., 2020	Opinião de especialista	Uma proposta dialógica para lidar com a incerteza.
Lee, 2020	Editorial	Um apelo por artigos sobre incerteza, motivado pela pandemia de covid-19.
McCarthy et al., 2020	Ensaio randomizado comparando estratégias de comunicação de incerteza diagnóstica	Intervenção educativa para comunicação da incerteza diagnóstica na unidade de emergência para melhorar a qualidade da assistência
Papanagnou et al., 2021	Estudo transversal com 287 alunos do 3º ano	Questionamentos aos alunos a fim de preparar treinamentos para lidar com a incerteza.
Romiti et al., 2021	Opinião de especialista	Argumentam que a pandemia de covid-19 intensificou nossa relação conflituosa com a incerteza.

### Seção 2: Arte

‘Arte’ foi o termo escolhido para incluir 87 artigos; é a maneira mais comum que os professores de medicina costumam ensinar. Baseia-se principalmente em discussões de casos e aprendizado da arte da medicina em contextos específicos, sem uma regra geral, mas com várias pequenas regras contingentes. Esses casos clínicos podem ser reais ou imaginários, e podem ser usados jogos ou plataformas eletrônicas. Os alunos e os médicos devem ser educados para apreciar a relevância das narrativas da doença no processo de cuidado/cura. ^13^

Doze artigos foram classificados sob o termo ‘heurística’. As heurísticas permitem que nos envolvamos em tomadas de decisão em contextos em que há um conjunto de dados incompleto, usando um processo que pode também exigir o descarte deliberado de alguns dados. Para um clínico, o processo baseado em heurística refere-se à integração intuitiva dos achados clínicos. Esta descrição é análoga a uma caracterização intuitiva de um cenário, para o qual pode ser atribuída uma probabilidade subjetiva que seria inserida no PCRB.

A arte desse “reconhecimento de padrões” ou de “semelhanças” é em grande parte inconsciente, sem esforço e, embora geralmente associada ao viés de disponibilidade e confirmação, é considerada eficiente. ^14^ Várias dimensões do raciocínio são consideradas, incluindo emoções. Essa “arte” contribui para a humanização da prática médica, forçando os médicos a pensar também em termos não técnicos. Neste “reconhecimento de padrão”, precisamos ir além da “vaga distinção entre o Sistema 1 e o Sistema 2 para modelos mais precisos de tomada de decisão diagnóstica”. ^15^ Em geral, os médicos estão aprendendo a pensar a partir de experiências anteriores em casos semelhantes. Logo, esse é um tipo de raciocínio indutivo, que não garante a verdade da conclusão, e pode ser traduzido em uma língua Bayesiana, como será argumentado na seção Discussão. A Tabela 2 mostra os principais termos foram utilizados nesses artigos como forma de caracterizar o raciocínio, mas não são uma regra de raciocínio.

**Tabela 2:** Palavras-chave dos artigos que discutem o raciocínio clínico como “arte”

Aprendizagem baseada em casos; Heurística; Processamento de informação; Lista de vieses cognitivos; Lista de habilidades; Memorização; Perspectiva filosófica; Modelos de papéis ( *Role models* ); Jogos sérios ( *Serious games* ); Valores; Ambiguidade; Pedindo ajuda; Escolhendo com sabedoria ( *Choosing wisely* ); Trilha de educação clínica ( *Clinical education track* ); Custos; Emoções; Teoria encapsulada ( *Encapsulated theory* ); Percepções gerais; Grau de prioridades de urgências; Sentimentos viscerais ( *Gut feelings* ); Uso de literatura não médica; Prevenção; Teatro realista; Achados clínicos salientes; Familiaridade e semelhança; tempo de reflexão

### Seção 3: A ideia geral do raciocínio

Estes 43 artigos foram agrupados por expressarem ideias mais gerais com algumas regras de raciocínio. Nesta seção, discutem-se dois tipos de processos de raciocínio necessários ao pensamento crítico: o indutivo e o dedutivo. São processos diferentes, apropriados para diferentes tipos de tarefas. ^16^ Apenas três artigos apresentam uma defesa mais explícita do pensamento dedutivo (hipotético-dedutivo, segundo o qual os dados obtidos geram hipóteses que são testadas na busca de confirmação ou de falsificação). Dois artigos fazem comparações qualitativas ^16^ ou quantitativas (em relação à validade e similaridade) ^17^ entre dedução e indução.

Por outro lado, 13 artigos apresentam uma defesa mais explícita do pensamento indutivo e probabilístico, aplicando o PCRB na tomada de decisão. Esta forma de pensar não se aplica apenas ao diagnóstico, mas também à interpretação de resultados de ensaios clínicos, ^18^ pois é uma forma geral de raciocínio. ^19^

Doze estudos foram agrupados na categoria MBE. Consideramos que a MBE é o julgamento clínico que envolve o conhecimento de noções metodológicas sobre delineamento de estudos e especialmente sobre noções probabilísticas sobre a diferença entre valor relativo e valor absoluto, grau de relevância clínica, impacto da intervenção (tamanho do efeito), habilidade de interpretação do intervalo de confiança dos resultados de um estudo em detrimento do valor isolado da significância estatística (valor de p), tomada de decisão terapêutica com base em NNT (número de pessoas necessário tratar para evitar um desfecho relevante) e ganho de sobrevida, análises de custo-efetividade, interpretação de meta-análises, além de noções básicas de mecanismos de busca de artigos e critérios de qualidade metodológica. A incorporação dessas informações ao PCRB, contendo maior ou menor probabilidade, requer a inserção desses conhecimentos de MBE na tomada de decisão. Eles funcionam como conhecimentos que avaliam de forma probabilística e indutiva o cenário de decisão que é modificado a cada nova informação. Uma nova evidência é assim incorporada ao PCRB como nova informação.

Dentre os 12 estudos restantes, cinco foram classificados como “esquemas indutivos”, um foi classificado como “métodos de escores de comparação de raciocínios” e sete classificados como “outros” que discutem questões relacionadas à importância do contexto (que é uma forma de avaliação do cenário inicial), pressupostos epistemológicos, ou ferramentas de caracterização das informações ou de discussões sobre o raciocínio não enquadradas nem como indutivas e nem como dedutivas, chamadas pelos autores de “métodos analíticos”, “polifonia”, “história e tendências”, “análise de decisão”.

## Discussão

Nesta seção, fazemos uma breve discussão dos resultados da revisão e apresentamos a proposta PCRB. A revisão sugere que a incerteza é ubíqua na medicina e que predomina o aprendizado por casos (indução). Observou-se uma frequência maior de raciocínios indutivos, probabilísticos e especialmente sobre o PCRB para tomada de decisão em comparação com o pensamento hipotético-dedutivo.

Tanto a lógica dedutiva (escassamente encontrada na revisão) como o pensamento indutivo (amplamente defendido na revisão) são expressões linguísticas, manipulação de signos. Os signos dedutivos usuais levam a pensamentos em categorias, do tipo V ou F, 0 ou 1, e nossa proposta é usar valores entre 0 e 1, o que pode ser ensinado. A motivação para a construção de linguagens lógicas formais foi a separação dos bons argumentos versus argumentos ruins; entretanto, é possível que a lógica não tenha nada a ver com processos mentais. ^6^ Por questões de viabilidade, a revisão foi restrita a descritores MeSH como tópicos principais ([majr]) na pesquisa reprodutível, o que a torna mais restritiva e menos sensível. Contudo, como não foi realizada meta-análise, considera-se satisfatória esta amostra da literatura sobre o tema para uma avaliação crítica.

O raciocínio indutivo envolve processamento de informações de baixo para cima, ou seja, das evidências para a teoria. A estratégia de processamento de dados é orientada por dados (validados, apropriados, não estruturados). Trata-se de um caminho exploratório de como chegar a uma conclusão, coletando evidências de casos e construindo um princípio geral. No pensamento indutivo, uma conclusão pode ser falsa mesmo que todas as premissas sejam verdadeiras (i.e., não garante a verdade da conclusão). É necessário reconhecer padrões e conexões, com a finalidade de formulação de hipóteses e teorias. ^16^ Por sua vez, o raciocínio dedutivo acontece de cima para baixo, das hipóteses (ou teorias) para as evidências: do conhecimento teórico sobre uma síndrome, procurar sinais e sintomas no paciente. Ou, quando a dedução não se dá das hipóteses para as evidências, se dá de uma hipótese para outra, como implicação das próprias hipóteses. A partir de uma suspeição diagnóstica, procura-se, no paciente, sinais e sintomas que confirmam a hipótese. Na dedução, uma conclusão não pode ser falsa se as premissas forem verdadeiras, tentando fazer previsão de consequências a partir das hipóteses ^16^ e não dos dados observacionais.

Um estudo sugere que médicos em treinamento e especialistas eventualmente geram hipóteses diagnósticas logo no início da investigação, e, assim, é provável que a coleta e interpretação subsequente de sinais clínicos sejam guiadas por essas hipóteses precoces ou precipitadas. Esta é uma importante fonte de erros no pensamento hipotético-dedutivo. Isso representa um desafio a educadores e pesquisadores médicos para elaborar estudos ou intervenções destinadas a reduzir erros. ^20^

**Figura 2:**
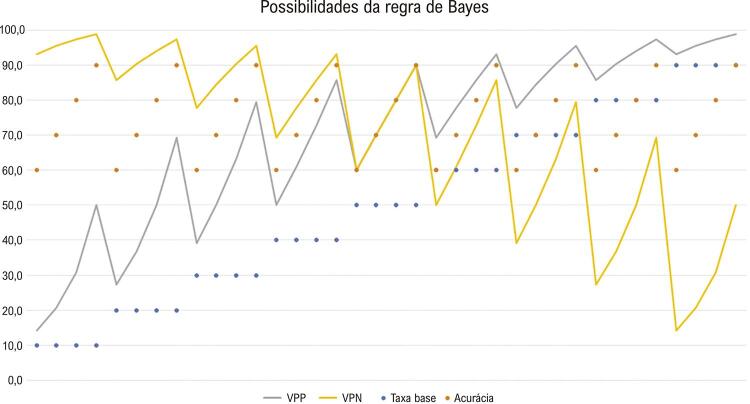
Probabilidades de resultado da regra de Bayes, baseada em graus de crença; Valores percentuais no eixo y. VPP: valor preditivo de um resultado positivo da nova informação; VPN: valor preditivo de um resultado negativo da nova informação. PCRB é baseado na taxa base do cenário prévio e na acurácia da nova informação ou da evidência. Subjetivamente, estima-se o grau de crença; objetivamente, busca-se evidências de boa qualidade. No raciocínio intuitivo, nas estimativas de taxa base muito elevadas ou muito baixas, a taxa base é determinante do raciocínio. Nas estimativas de taxa base intermediária, a acurácia é determinante do resultado do raciocínio. Explicação disponível em https://youtu.be/gdHM3pkwDHc.

A proposta PCRB sobre a probabilidade e o pensamento Bayesiano, com base na revisão de literatura filosófica ^5 , 7 , 8^ e corroborada pela revisão sistemática, permite uma linguagem unificada. Como ilustra a Figura 2 , sob uma perspectiva subjetiva (grau de crença) da probabilidade, o PCRB considera o cenário prévio e o resultado da investigação atual para estimar a probabilidade de o cenário ser mais apropriadamente interpretado após tal investigação.

O PCRB condiciona a interpretação da nova informação ao cenário prévio. Assim, o pensamento Bayesiano é uma inferência indutiva em duas etapas temporalmente articuladas. ^21^ Na caracterização do cenário inicial, todos os seus aspectos são considerados, e as teorias contidas na formação do médico vão influenciar. No entanto, é prioritário considerar aquilo que é observado, ou seja: primeiro a anamnese, depois o exame clínico, depois os exames complementares, com foco nos problemas do paciente. O risco de usar ingenuamente o pensamento hipotético dedutivo é, durante as fases iniciais da investigação, o médico começar a procurar, no paciente, confirmações de coisas que estão em sua mente (uma espécie de viés de confirmação). O risco de usar ingenuamente o PCRB é acreditar que as crenças ou as frequências observadas garantem a conclusão.

Por exemplo, insuficiência cardíaca é uma síndrome de difícil diagnóstico em suas formas leves ou com comorbidades pulmonares. O médico, durante a avaliação de um paciente com uma forma leve de insuficiência cardíaca, não se sabe se o paciente tem ou não tem a doença. Começamos a investigação sem saber, então não é um conhecimento que funda o diagnóstico, seu fundamento é o “não saber”, é a incerteza; e sua construção é orientada pelo cenário inicial (com foco nos sinais e sintomas e não com base na hipótese precoce) e revisada pelos exames complementares (gerando cenários pós-teste). O pensamento hipotético dedutivo pode ser útil, mas seu risco é usar um conjunto muito restrito de hipóteses, sem considerar todas as hipóteses alternativas. Os sintomas relatados e sinais observados são a base inicial, o cenário pré-teste do raciocínio durante a consulta.

Pode ser contra argumentado que a teoria sobre a insuficiência cardíaca fornece um modelo análogo aos modelos e estruturas da lógica dedutiva, e os critérios para o diagnóstico no paciente seriam semelhantes a uma dedução, avaliando se as proposições e inferências satisfazem ao modelo (padrão típico de raciocínio dedutivo). Contudo, o raciocínio clínico parte do indivíduo contingente em seu contexto para as hipóteses ou para a teoria. São os sintomas e sinais e a sequência de exames que devem conduzir a formulação das hipóteses e não as hipóteses conduzirem o raciocínio. As evidências encontradas no exame clínico e as informações obtidas na anamnese levam o médico a um raciocínio que é indutivo e não dedutivo. É a partir das “pistas” (sintomas, sinais, exames) que se elabora hipóteses e isto inclui, neste exemplo, não apenas a hipótese da insuficiência cardíaca bem como outras hipóteses para explicar as pistas. O médico astuto deverá pensar em hipóteses alternativas, construídas a partir dos dados observados no paciente e não em uma lista de diagnósticos diferenciais de um livro que não considera o contexto individual.

O mesmo vale para o prognóstico: é a partir do paciente, contingente em seu contexto, que o médico tenta estimar um prognóstico. Mesmo que iluminado pelos estudos, são os dados do indivíduo que devem entrar na análise para se determinar em qual estudo ou subtipo populacional aquele paciente se encaixa. O mesmo vale para o tratamento: deve-se analisar o perfil clínico do paciente, em qual momento do espectro da doença ele se encontra e em qual contexto para decidir quais evidências de estudos terapêuticos melhor se encaixam ao paciente. Não se devem encaixar as diretrizes no paciente de forma acrítica, e sim avaliar em qual perfil do espectro clínico o paciente se encontra nas diretrizes.

Para procurar soluções para os problemas, valores e preferências do paciente, o uso da literatura médica segue a mesma regra: as ferramentas descritas na linguagem da MBE devem considerar como prioritárias as estimativas quantitativas e absolutas (não relativas) das intervenções diagnósticas, prognósticas ou terapêuticas, com base em dados empíricos e não meramente teóricos, na construção de uma hierarquia Bayesiana. Não basta um estudo, uma evidência. Há muitas situações de incerteza na aplicação prática das evidências e no grau de acurácia de cada evidência. Há uma hierarquia que precisa ser respeitada. Trata-se de um arcabouço que sustenta a tomada de decisão. A decisão é tomada sob algum grau de incerteza residual, com base na maior probabilidade subjetiva, que incorpora dados objetivos dos estudos. No PCRB, entendendo ‘provável’ como ‘provável de ser um conhecimento mais acurado, mais útil’; há uma sequência hierárquica, apresentada na Tabela 3 .

**Tabela 3:** Hierarquia bayesiana no pensamento conforme a regra de Bayes

Opinião de especialista é mais provável que opinião de não especialista. Opinião é menos provável que argumento. Argumento de especialista é mais provável que argumento de não especialista. Argumento é menos provável que evidência. Evidência com método confiável é mais provável que evidência com método menos confiável. Evidência com método confiável produzida por pessoas com menos conflitos de interesse é mais provável que evidência produzida por pessoas com mais conflitos de interesse. O passo 6 é mais provável se for verificado pelo mesmo método por outros pesquisadores que não aqueles do passo 6.

Sob o PCRB, sempre há um resíduo de incerteza, entretanto há uma redução da incerteza à medida que se acumulam contextos, cenários e evidências. Cada informação nesta hierarquia é incorporada ao contexto, como uma nova informação com determinada acurácia agregada a uma probabilidade prévia. Um diagnóstico, um prognóstico ou um tratamento são vistos como probabilísticos ou como hipóteses e não como “verdadeiros”. O discurso linguístico mais apropriado na comunicação ao paciente deve ser orientado pelo que parece mais provável à luz dos dados disponíveis naquele momento. O princípio da bivalência da lógica clássica, do verdadeiro ou do falso, é insuficiente para expressar a incerteza residual. O PCRB lida com a incerteza sem cair no relativismo.

## Conclusões

A revisão da literatura mostrou que: 1) a incerteza é a condição epistêmica para o raciocínio; 2) por isso, usamos predominantemente a probabilidade; 3) há muita confusão conceitual sobre o tema. O pensamento Bayesiano aqui proposto, e amplamente subsidiado pela revisão de literatura, é um pensamento probabilístico em duas etapas que pode ser ensinado. A regra de Bayes é uma ferramenta linguística, uma regra geral de raciocínio, de diagnóstico, de comunicação científica e de revisão do conhecimento médico conforme novas evidências. A caracterização do cenário inicial é uma arte que envolve múltiplos aspectos, sendo alguns subjetivos, mas que podem ser inseridos no PCRB, sob a luz da teoria subjetiva da probabilidade.
