# Supplementary material for: Dedução, Indução e a Arte do Raciocínio Clínico na Educação Médica: Revisão Sistemática e Proposta Bayesiana
Source: Arq Bras Cardiol. 2022 Nov 9;119(5 Suppl 1):27–34. [Article in Portuguese] doi: 10.36660/abc.20220405 (PMC9750195; doi:10.36660/abc.20220405)
Supplement: Translation

# Translation: Deduction, Induction and the Art of Clinical Reasoning in Medical Education: Systematic Review and Bayesian Proposal

**Authors:** Marcos Roberto de-Sousa, Túlio Roberto Xavier de Aguiar

**Affiliations:** 1 Brazil Hospital das Clínicas da Universidade Federal de Minas Gerais, Belo Horizonte, MG – Brazil; 2 Brazil Departamento de Filosofia da Faculdade de Filosofia e Ciências Humanas – FAFICH – Universidade Federal de Minas Gerais (UFMG), Belo Horizonte, MG – Brazil

**Keywords:** Education, Medical, Problem Solving, Learning, Clinical Decision-Making, Systematic Review, Bayes Theorem, Evidence Based Medicine

## Abstract

**Background:**

Clinical reasoning is at the core of medical practice and entangled in a conceptual confusion. The duality theory in probability allows to evaluate its objective and subjective aspects.

**Objectives:**

To conduct a systematic review of the literature about clinical reasoning in decision making in medical education and to propose a “reasoning based on the Bayesian rule” (RBBR).

**Methods:**

A systematic review on PubMed was conducted (until February 27, 2022), following a strict methodology, by a researcher experienced in systematic review. The RBBR, presented in the discussion section, was constructed in his undergraduate dissertation in Philosophy at Minas Gerais Federal University. Heart failure was used as example.

**Results:**

Of 3,340 articles retrieved, 154 were included: 24 discussing the uncertainty condition, 87 on vague concepts (case discussion, heuristics, list of cognitive biases, choosing wisely) subsumed under the term “art”, and 43 discussing the general idea of inductive or deductive reasoning. RBBR provides coherence and reproducibility rules, inference under uncertainty, and learning rule, and can incorporate those vague terms classified as “art”, arguments and evidence, from a subjective perspective about probability.

**Conclusions:**

This systematic review shows that reasoning is grounded in uncertainty, predominantly probabilistic, and reviews possible errors of the hypothetico-deductive reasoning. RBBR is a two-step probabilistic reasoning that can be taught. The Bayes theorem is a linguistic tool, a general rule of reasoning, diagnosis, scientific communication and review of medical knowledge according to new evidence.

## Introduction

Reasoning is at the core of medical practice, spread in several disciplines and traditions.^1^ Reasoning occurs through biochemical, electrical and magnetic processes that are not well understood despite advances in neuroscience, since it is pre-linguistic.^2^ By means of linguistic expression, we can teach several types of reasonings: logical, mathematical, and probabilistic reasoning, including the “reasoning based on the Bayesian rule” (RBBR).

“Evidence-based medicine” (EBM) describes a movement against an excessive dependence on clinical judgement and experience in treatment decision-making.^3^ EBM is the conscious, explicit and careful use of the best available evidence in the decision making about individual patient care.^4^ Under a dual perspective of probability, EBM would be a version that values the frequencies in clinical trials, and medical reasoning would be the coherent formation of “degrees of belief”,^5^ governed by the subjective theory of probability and the RBBR. This incorporates evidence into a previous context, so that a clinical trial alone is not able to overcome it.

Logical reasoning tends to be deductive and deterministic, and thus, different from probabilistic reasoning which is inductive and based on uncertainty (non-deterministic). We will carry out an attack against the abusive use of deductive reasoning and isolated evidence. Both evidence and deductive and argumentative reasonings can be incorporated into RBBR. The motivation of this work was a philosophical research^5 - 8^ about reasoning conducted by a physician experienced in teaching and research in health. The objective was to conduct a systematic review of the literature about reasoning and decision making in medical education, and to propose an explanation and arguments in favor of RBBR, a specific type of probabilistic reasoning.

## Methods

The systematic review was performed following PRISMA guidelines.^9^ In the MeSH (Medical Subject Headings) thesaurus, ‘clinical reasoning’ is a subheading of ‘diagnosis’, and ‘decision making’ is a subheading of ‘mental processes’. In this descriptor, ‘decision making’ is defined as “the process of making a selective intellectual judgment when presented with several complex alternatives consisting of several variables, and usually defining a course of action or an idea”. ‘Medical education’ was the main question of interest and thus the following reproducible search was carried out:

(((“Education, Medical”[Majr]) AND ((“Clinical Reasoning”[Majr]) OR “Decision Making”[Majr]))) AND ((“1952/02/27”[Date - Entry]: “2022/02/27”[Date - Entry]))

The references of the articles were also used. Inclusion criteria were articles written in English, German, Portuguese, and Spanish; all types of publications focusing on education for clinical reasoning, decision making, methods of thinking, case studies. Exclusion criteria were articles on decision-making on choices in the medical career, medical marketing, healthcare, and other aspects not related to reasoning. Comparisons using questionnaires for specialists, questionnaires for students, results of structured cases, schemes or serious games were only included if arguments about general rules of reasoning were discussed in the study.

The critical assessment of medical literature included the use of the RBBR,^10^ as this was an “a priori” proposal. After screening the articles based on their titles and abstracts using the RAYYAN application,^11^ articles were selected after the first reading of full text. Since the main interest lay on the authors’ arguments in favor of clinical reasoning, all articles were read for a second time for analysis of the arguments and were divided into three groups defined *a posteriori* : 1- uncertainty; 2- vague concepts subsumed under the term “art”; and 3- general idea of reasoning. Although this last group was of the greatest interest, the other two were considered for providing relevant arguments.

The RBBR proposal was elaborated by a physician researcher in his undergraduate dissertation in Philosophy, supervised by an experienced science philosopher. In this work, this physician evaluated the work by Ian Hacking^5 , 8^ and Donald Gillies^7^ about the philosophical theories about probability and the Bayes’ theorem. This is a conditional probability of a posterior hypothesis due to evidence, i.e., it is a revision of existing probabilities given new evidence or information. The theorem is expressed as:


$$\mathrm{Pr}(H|E)=\frac{\mathrm{Pr}(H)\mathrm{Pr}(E|H)}{\mathrm{Pr}(H)\mathrm{Pr}(E|H)+\mathrm{Pr}(∼H)\mathrm{Pr}(E|∼H)}$$

Or as: “ ***post-test odds = likelihood ratio x pre-test odds*** ”. Sensitivity, specificity and likelihood ratio are alternatives to accuracy measurement.^12^ Pr(E|H) stands for sensitivity and Pr(E|~H) = (1 – specificity). Pr(H|E) is the revision of the hypothesis (post-test probability or probability of posterior scenery) considering the base rate (Pr(H)) of the previous probability (pre-test probability) and the accuracy of the new evidence or information. Posterior probability is determined by prior probability (base rate) and accuracy (also a probability) of the new evidence or information. This is a conditional combination of probabilities in two temporally integrated steps. Based on the subjective theory of probability, the physician’s state of belief is updated based on information collected from medical history taking, clinical examination, complementary tests and from medical literature (which may provide less subjective measures). In the discussion section, heart failure is used to illustrate possible uses of RBBR in the diagnosis, prognosis, and therapeutic choices.

## Results

Figure 1 illustrates the flowchart of article selection. Of 3,340 references analyzed, 154 articles were included: 24 discussing uncertainty, 87 on concepts subsumed in the term “art”, and 43 discussing the general ideal of reasoning. These three groups of articles will be presented in three sections. Due to lack of space, it would be unfeasible to cite 154 references in this article; for this reason, an appendix with a brief explanation about the classification and division of the articles into groups is provided (https://bit.ly/3EMx5sp).

**Figure 1:** Flowchart of study selection.

### Section 1: Uncertainty

There were 24 articles on the concept that medical students and physicians should learn how to deal with ‘uncertainty’ ( Table 1 ). The meaning of the word “uncertainty” includes from diagnostic uncertainty to uncertainty about physician’s knowledge and the scientific literature itself. These articles were grouped to form the epistemic basis of RBBR. Uncertainty is a human condition for contingent reasoning. Instead of rejecting it, physicians should understand and learn to deal with uncertainty, by means of arguments and evidence, in a hierarchy that will be presented in the final proposal.

**Table 1:** Main arguments or ideas described in the 24 articles on uncertainty

Author, Year	Type of publication	Main argument
**Whitehorn, 1963**	Expert’s opinion	Against determinism, in favor of probability and values.
Elstein, 1982	Editorial	A proposal to deal with uncertainty: “More time is spent on the computation and interpretation of chi-squares, T-tests, and other inferential techniques than on the statistics of opinion revision and decision making – the so-called Bayesian approach – though the latter is more relevant to the daily work of clinical practice (…). Clinical practitioners are properly more concerned with the soundness of decisions made in particular circumstances than with the soundness of general inferences. For this purpose, the Bayesian outlook will be more helpful, and instruction in the logic of clinical decisions should incorporate it.”
Gunderman, 2005	Expert’s opinion	A dialogic proposal to deal with uncertainty.
Nevalainen et al., 2009	22 students' reflective learning diaries	Reflective writing as a proposal to express and deal with uncertainty.
Blanch et al., 2009	147 interactions between medical students and patients	The authors found a negative perception of medical students who expressed uncertainty to patients. Types of sentences were analyzed.
Charlin et al., 2010	Panel with experts, residents and students	Standardization methods to compare scores of individual examiners with those of a reference panel to deal with uncertainty.
Schwartz, 2011	Expert’s opinion	A proposal to teach decision making as the main question in medicine.
Hamui-Sutton et al., 2015	128 Residents: interview and expert’s opinion	A comprehensive evaluation of several types of uncertainty in medical practice.
Niedermier, 2016	Letter to the editor	Points to the negative effects of uncertainty and the need to deal with it.
Simpkin et al., 2016	Expert’s opinion	Speak about “hypotheses” rather than “diagnoses”, embracing uncertainty as an attitude.
Cooke et al., 2017	594 trainees	Uncertainty stress and reluctance in communicating uncertainty.
Cooke et al., 2017	Expert’s opinion	A proposal to embrace uncertainty and accept more than one solution to a problem.
Oferta, 2017	Expert’s opinion	Literature, music, art and humanities to learn to deal with uncertainty.
Kim et al., 2018	Review	Proposal of strategies to manage uncertainty.
Simpkin et al., 2018	86 Interviews with residents and expert’s opinion	High levels of uncertainty stress and low levels of resilience seem to be associated with depression and burnout.
Tonelli et al., 2019	Expert’s opinion	Philosophical approach of uncertainty, including metaphysics, fallibilism, and epistemological reasoning.
Davidson, 2019	Editorial	A set of recommendations to deal with uncertainty in medical science: “Authors should be appropriately tempered in their conclusions, using language that acknowledges uncertainty where appropriate. The conclusions should be influenced by not only the P value but also the effect size and bounds of the 95% confidence intervals."
Ying et al., 2019	70 residents	The study suggested that individuals that are more comfortable with uncertainty can experience greater satisfaction at work.
Stephens et al., 2020	Qualitative study with 608 students	Motivate medical educators to incorporate aspects of tolerance to uncertainty in academic and learning environments.
Beck et al., 2020	Expert’s opinion	A dialogic proposal to deal with uncertainty.
Lee, 2020	Editorial	A call for articles on uncertainty motivated by the COVID-19 pandemic.
McCarthy et al., 2020	Randomized trial comparing communication strategies in disclosing diagnostic uncertainty	Educational intervention for clear communication about diagnostic uncertainty to improve quality of care at the emergency department.
Papanagnou et al., 2021	Observational cross-sectional study with third-year medical students	The students were surveyed for the development of trainings to deal with uncertainty.
Romiti et al., 2021	Expert’s opinion	The authors argued that the COVID-19 pandemic intensified our conflicting relationship with uncertainty.

### Section 2: Art

‘Art’ was the term chosen for inclusion of 87 articles. It is the most common teaching method used by medical teachers. The method is mainly based on case discussions and learning of the art of medicine in specific contexts, without a general rule, but rather with several small contingent rules. These clinical cases may be either real or imaginary, and electronic games or platforms may also be used. Medical students and physicians should be educated to appreciate the relevance of narratives of disease in the care process.^13^

Twelve articles were classified within the term “heuristic”. Heuristics allow us to be involved in decision making in missing data situations, by a process that may also requires a deliberate disposal of data. For the clinician, the process based on heuristic refers to an intuitive integration of clinical findings. This description is analogous to an intuitive characterization of a scenario, to which a subjective probability that would be inserted to RBBR may be attributed.

The art of this “standard recognition” or “similarities” is largely unconscious, effortless and, despite usually associated with availability and confirmation biases, it is considered efficient.^14^ Several thinking dimensions are considered, including emotions. This “art” contributes to humanization in medical practice, forcing physicians to also think in non-technical terms. In this “standard recognition”, we must go beyond the “vague distinction between System 1 and System 2 towards more precise models of diagnostic decision making.”^15^ In general, physicians have been learning to think from prior experiences in similar cases. Therefore, this is a type of inductive reasoning, that does not guarantee the truth of conclusion and may be translated into a Bayesian language, as described in the Discussion section. Table 2 shows the main terms in these articles used to characterize reasoning, but not to establish reasoning rules.

**Table 2:** Keywords of articles that discuss clinical reasoning as “art”

Case-based learning; Heuristic; Information processing; List of cognitive biases; List of abilities; Memorization; Philosophical perspective; Role models; Serious games; Values; Ambiguity; Asking for help; Choosing wisely; Clinical education track; Costs; Emotions; Encapsulated theory; General perceptions; Urgent priority degree; Gut feelings; Use of non-medical literature; Prevention; Realistic theater; Salient clinical findings; Familiarity and similarity; Reflection time

### Section 3: General rule of reasoning

These 43 articles were grouped for expressing more general ideas with some reasoning rules. In this section, two types of reasoning processes needed to critical thinking are discussed: the inductive and deductive processes; these are different processes, appropriate to different types of tasks.^16^ Only three articles presented a more explicit defense of the deductive thinking (hypothetic-deductive, by which the data obtained generate hypothesis that are tested in search of confirmation or falsification). Two articles make qualitative^16^ or quantitative comparisons (in relation to validity and similarity)^17^ between deduction and induction.

On the other hand, 13 articles present a more explicit defense of an inductive and probabilistic thinking, applying the RBBR in the decision making. This way of thinking is applied not only to diagnosis, but also to interpretation of clinical trials’ results,^18^ as this is a general form of reasoning.^19^

Twelve articles were grouped in the EBM category. We consider that EBM is the clinical judgement that involves knowledge of methodological notion about study design, and especially about probabilistic notion about the difference between relative and absolute values, clinical relevance degree, intervention impact (effect size), interpretation of confidence intervals of a study results rather than isolated values of statistical significance (p-value), therapeutic decision making based on NNT (number of individuals needed to treat to prevent a relevant outcome) and survival gain, cost-effectiveness analysis, interpretation of meta-analyses, in addition to basic notions of article search mechanisms and methodological quality criteria. Incorporation of this information into RBBR, with higher or lower probability, requires the inclusion of these knowledges of EBM into decision making. They act as knowledges that evaluate, in a probabilistic and inductive way, the decision scenario that is modified by each new piece of information. New evidence is then incorporated into RBBR as new information.

Among the 12 remaining studies, five were classified as “inductive schemes”, one as “score methods for reasoning comparison” and seven classified as “others” that address issues related to the importance of the context (which is a method to evaluate the initial scenario), epistemological assumptions, or tools for characterization of information or discussions about reasoning, not categorized neither as inductive nor deductive, named by the authors as “analytical methods”, “polyphony”, “histories and trends”, “decision analysis”.

## Discussion

In this section, we will make a brief discussion about the results of this review and present the RBBR proposal. The review suggests that uncertainty is ubiquitous in medicine, and case-based learning is predominant (induction). There was a higher frequency of inductive, probabilistic reasoning and especially RBBR for decision making as compared with the hypothetico-deductive approach.

Both deductive logic (scarcely found in the literature) and inductive reasoning (widely defended in this review) are linguistic expressions, manipulations of signs. Usual deductive signs lead to categorical thinking (e.g.: T or F, 0 or 1) and our proposal is to use values between 0 and 1, which can be taught. The motivation for the construction of formal logical languages was the differentiation of good *versus* bad arguments; however, it is possible that logic has nothing to do with mental processes.^6^ For feasibility reasons, this review was restricted to MeSH descriptors as the main topics ([majr]) in the reproductive research, which makes it more restrictive and less sensitive. Nevertheless, as a meta-analysis was not performed, this literature sample can be considered satisfactory for a critical analysis of the theme.

Inductive reasoning involves information processing in a bottom-up approach, *i.e.* , from evidence to theory. The strategy of data processing is driven by data (validated, appropriate, unstructured). This is an exploratory pathway to get to a conclusion, collecting evidence of cases and constructing a general principle. In the inductive thinking, a conclusion may be false even when all premises are true ( *i.e* ., does not guarantee the truth of the conclusion). It is necessary to recognize patterns and connections to formulate hypotheses and theories.^16^ Deductive reasoning, in turn, occurs in a top-down approach, i.e., from hypotheses (or theories) to evidence: from theoretical knowledge about a syndrome, to examine the patient for signs and symptoms. Alternatively, when deduction does not occur from hypotheses to evidence, it occurs from one hypothesis to another one, as implication of the own hypotheses. From diagnostic suspicion, signs and symptoms are sought to confirm the hypothesis. In deduction, a conclusion cannot be false if premises are true, in attempt to predict the consequences not from observational data, but rather from the hypotheses.^16^

One study suggests that training physicians and specialists eventually generate diagnostic hypotheses in the beginning of the investigation and, therefore, it is likely that the collection and subsequent interpretation of clinical signs are guided by these early hypotheses. This is an important source of errors in the hypothetico-deductive reasoning. It represents a challenge to medical educators and researchers to develop studies or interventions aimed at reducing errors.^20^

The RBBR proposal about probability and the Bayesian theorem, based on the review of philosophical literature^5 , 7 , 8^ and corroborated by the systematic review, allows a unified language. As illustrated in Figure 2 , from a subjective perspective (degree of belief) of probability, RBBR considers both the prior scenario and the result of current investigation to estimate the likelihood of the scenario be more appropriately interpreted after such investigation.

**Figure 2:** Probability of results in the Bayes theorem based on the degree of belief; percentage in the y-axis; PPV: predictive value of a positive result of the new information; NPV: predictive value of a negative result of the new information; the Bayesian reasoning is based on the baseline rate of the prior scenario and on the accuracy of the new information or evidence. Subjectively, the degree of belief is estimated and, objectively, good-quality evidence is searched. In the intuitive reasoning, in the estimates of either very low or very high degree of belief, the baseline rate is determinant of reasoning. In the estimates of intermediate baseline rate, the accuracy is determinant of the reasoning result. The explanation is available at https://youtu.be/EVqfyUNe-bU

RBBR can condition the interpretation of new information on the prior scenario. Thus, the Bayes theorem consist of an inductive inference with two temporally articulated steps.^21^ In the characterization of the initial scenario, all aspects are considered, and the theories related to medical formation can be influent. However, it is paramount to observe: first the medical history, then clinical examination, followed by complementary tests focusing on patients’ problems. The risk of naive use the deductive hypothetical thinking is that, during the initial steps of the investigation, the physician starts to search for confirmation of things that are going on in their heads (a type of confirmation bias). The risk of naive use the RBBR is to believe that beliefs or the observed frequencies will guarantee the conclusion.

For example, heart failure is a syndrome that is difficult to be diagnosed in its mild forms or in case of pulmonary comorbidities. During examination of a patient with mild heart failure, it is not known whether the patient has or not the disease. We start the investigation without this information; therefore, this knowledge is not the ground of the diagnosis, whose foundation is the “not knowing”, the uncertainty, and whose construction is guided by the initial scenario (focused on signs and symptoms rather than on prior hypothesis) and revised by complementary tests (generating post-tests scenarios). Hypothetical-deductive reasoning may be useful, but its risk lies in using a very limited set of hypotheses, without considering alternative ones. Symptoms reported and signs observed are the initial basis, the pre-test scenario of reasoning during consultation.

A counterargument to this hypothesis may be that the theory of heart failure provides a model analogous to the models and structures of deductive logic, and that the diagnostic criteria would be similar to deduction, evaluating whether propositions and inferences would satisfy the model (typical standard of deductive reasoning). However, clinical reasoning occurs from contingent individuals in their contexts to hypotheses or to the theory. Symptoms and signs in addition to the series of tests should guide hypothesis formulation, and not the hypotheses per se guide the reasoning process. Evidence obtained from clinical examination and information obtained from medical history lead the physician to an inductive rather than deductive reasoning. From these “hints” (symptoms, signs, tests), the physician makes hypotheses, not only of heart failure in this example, but also other possibilities to explain the hints. An astute physician must think about alternative hypotheses, constructed from data observed in the patient and not from a list of differential diagnoses from a book that does not consider individual contexts.

The same works with prognosis: physicians should estimate it based on patient’s condition and context. Even enlightened by studies, physicians must analyze patient’s individual data to decide the type of study or population subgroup that the patient should be included. The same works for treatment: patient’s clinical profile, patient’s context, and stage of the disease spectrum should be evaluated to decide which evidence from therapeutic studies best fit the patient. Guidelines should not be applied uncritically, but rather, patient’s clinical spectrum should be scrutinized to determine where it better fits into the guidelines.

When seeking for solutions to patient’s problems, values and preferences, the use of medical literature should follow the same rule: the tools described in the EBM language should prioritize quantitative and absolute (not relative) estimates of diagnostic, prognostic or therapeutic interventions, based on empirical (and not merely theoretical) data, for the construction of a Bayesian hierarchy. Only one study or one evidence is not enough. There is a whole framework that supports the decision making. A decision is made under some degree of residual uncertainty, based on the highest subjective probability, that incorporates objective data from the studies. In the RBBR, “likely” is taken as “likely to be a more accurate, more useful knowledge”; there is a hierarchical sequence, as described in Table 3 .

**Table 3:** Bayesian hierarchy in the Bayesian reasoning

1An expert opinion is more likely than a non-expert opinion. Opinion is less likely than argument. An expert argument is more likely than a non-expert argument. Argument is less likely than evidence. Evidence with a reliable method is more likely than evidence with a less reliable method. Evidence with a reliable method produced by individuals with less conflict of interest is more likely than evidence produced by individuals with more conflict of interest. Step 6 is more likely if checked using the same method by investigators other than those of the step 6.

In the RBBR, there is always residual uncertainty, but uncertainty reduces with increasing contexts, scenarios and evidence. Each piece of information of this hierarchy is aggregated into the context, as new information with certain accuracy incorporated into a previous probability. A diagnosis, a prognosis or a treatment can be seen as probabilistic or as hypotheses rather than as “truth”. The most appropriate linguistic discourse in patient communication should be based on what seems to be the most likely considering the available data at that moment. The principle of bivalence of classical logic, of “true or false”, is insufficient to explain residual uncertainty. RBBR deals with uncertainty without falling into relativism.

## Conclusion

This literature review demonstrated that: 1) uncertainty is an epistemic condition of reasoning; 2) for this reason, probability is predominantly applied; 3) there is considerable conceptual confusion about the subject. RBBR, here proposed, and strongly supported by the literature review, is a two-step probabilistic reasoning that can be taught. Bayes theorem is a linguistic tool, a general rule of reasoning, diagnosis, scientific communication and review of medical knowledge according to new evidence. Characterization of the initial scenario is an art that involves multiple aspects, some of them subjective, but that can be inserted into the RBBR, under the light of the subjective theory of probability.
